# Fall risk and falls count by functional status in patients with hip fracture

**DOI:** 10.1186/s12877-026-07628-y

**Published:** 2026-05-09

**Authors:** Seigo Inoue, Yohei Otaka, Michiyuki Kawakami, Shin Kitamura, Kunitsugu Kondo

**Affiliations:** 1 Department of Rehabilitation Medicine, Tokyo Bay Rehabilitation Hospital, Narashino, Chiba Japan; 2 Department of Rehabilitation Medicine, School of Medicine, Fujita Health University, 1-98 Dengakugakubo, Kutsukake, Toyoake, Aichi 470-1192 Japan; 3 Department of Rehabilitation Medicine, Keio University School of Medicine, Shinjuku, Tokyo Japan

**Keywords:** Activities of daily living, Hip fracture, Falls, Fall prevention, Inpatients, Rehabilitation

## Abstract

**Background:**

Patients with hip fractures are at high risk of falling. In hospitals, identifying high-risk patients based on their capabilities and implementing targeted prevention strategies is essential. Because fall risk changes with functional recovery, it should be assessed longitudinally rather than at a single time point. This study aimed to determine whether the risk of falls (falls per 1,000 person-days) was stratified by motor and cognitive functional status and to examine the relationship between fall incidence rates and the actual number of falls in each functional status.

**Methods:**

This retrospective observational study included 824 consecutive patients with hip fractures admitted to a rehabilitation hospital. Data on falls during hospitalization and Functional Independence Measure (FIM) scores were retrieved from medical records. Average FIM scores for the motor and cognitive items were obtained and categorized into complete dependence, modified dependence, and independence. Fall rates and number of observed falls in each combined condition were investigated.

**Results:**

The highest fall rate was observed when patients were in states of modified motor dependence and complete cognitive dependence (11.4 falls/1,000 person-days; 17 falls; 9.3% of all falls). In contrast, patients in independent motor and cognitive states had a lower fall rate (2.0 falls/1,000 person-days) but accounted for a larger proportion of total falls (32 falls; 17.6% of all falls), representing 1.9 times the total number of falls observed in the highest fall-rate group.

**Conclusion:**

This study successfully demonstrated that fall risk in patients with hip fractures varies according to functional status, peaking during phases of modified motor dependence and complete cognitive dependence. However, owing to longer observation periods, a greater number of falls occurred among those in low-risk states than among those in high-risk states, demonstrating the “prevention paradox.” Effective management requires a dual strategy: intensive interventions targeting high-risk phases and standardized preventive measures for all patients to address cumulative risk during the recovery process.

## Background

Patients hospitalized for hip fractures constitute a uniformly high-risk population for falls. In general, a history of falls is the strongest predictor of falls [1, 2]. In rehabilitation hospitals, 12–39% of the patients with hip fractures have been reported to experience a fall [3–5]. Furthermore, the recurrence rate of falls after hip fractures is reported to be 53% within 6 months [6] and as high as 56% within 1 year [7]. According to the World Guidelines for falls prevention and management in older adults [8], older adults admitted primarily for a fall are considered to be at high risk of recurrent falls. Therefore, patients admitted for the treatment of fall-related injuries, such as hip fractures, are a group for whom appropriate and targeted fall prevention measures should be prioritized.

In hospital settings, the fundamental strategy for fall prevention has traditionally relied on risk stratification to guide the allocation of limited preventive resources [9–11]. However, recent World Guidelines [8] and numerous studies [12–18] have questioned the effectiveness of conventional score-based fall risk screening tools. These tools have been reported to demonstrate insufficient diagnostic accuracy to warrant routine use [12–17], and their contribution to reducing fall rates relative to the clinical workload has been considered limited [19, 20]. One reason is that many conventional screening tools are based on single point-in-time assessments on admission, thus possessing inherent structural limitations. Specifically, they may not adequately capture the dynamic changes in fall risk that occur as functional status improves during the recovery process. To address these limitations, it is essential to employ assessment methods that can track shifts in functional status throughout the rehabilitation period. Internal risk factors for falls are often characterized by physical functions and cognitive abilities [21–23]. In rehabilitation settings, the Functional Independence Measure (FIM) [24] serves as a standardized indicator of patients’ functional status. Because FIM reflects changes in motor and cognitive abilities over time, it may be suitable for capturing temporal changes in fall risk during inpatient rehabilitation. Previous studies [25–27] have suggested that FIM-based assessments may demonstrate predictive performance comparable to, or even exceeding, that of traditional fall risk screening tools. Furthermore, research has shown that fall rates tend to increase when patients reach a “moderate” level of functional status during the recovery process [28], suggesting that FIM may reflect temporal changes in fall risk. Identifying combinations of motor and cognitive function states (FIM score combinations) associated with a high risk of falls may be useful for optimizing preventive measures. Rather than relying solely on single assessments on admission, focusing on these high-risk functional stages may allow for a more efficient allocation of limited prevention resources.

In hospital settings, fall risk is primarily defined by the “fall rate,” which represents the frequency of events relative to a specific observation period. However, effective fall countermeasures require two distinct perspectives: fall prevention, which focuses on reducing the risk level (the rate), and fall management, which aims to reduce the total number of falls. These two metrics do not necessarily align. This is because the total number of falls is calculated by multiplying the fall rate by the observation time. Therefore, the number of falls is affected not only by the fall rate (risk of falls) but also by the length of observation in that risk condition. A study [29] of patients with stroke admitted to a rehabilitation hospital showed that the number of falls was not necessarily high in functional states with high fall rates, and more falls were observed in functional states with low fall rates. This gap highlights the current limitations in fall prevention management, particularly the lack of clarity about which patients should be prioritized for preventive measures. Owing to limited hospital resources, implementing comprehensive and targeted fall prevention strategies for all patients is not feasible.

This study aimed to determine whether fall risk can be stratified by combinations of motor and cognitive functional states, as measured via the FIM, and to analyze the relationship between these risk levels and the actual frequency of falls in hospitalized patients with hip fractures. We hypothesized that a clear gradient in fall risk exists according to combinations of functional states and that, consistent with previous findings [29], falls among patients in the highest-risk state would not necessarily account for the majority of all falls.

## Methods

### Study design and participants

Ethical approval for this retrospective observational study was obtained from the Institutional Review Board of Tokyo Bay Rehabilitation Hospital, Japan (approval number: 230-5). The study was conducted at Tokyo Bay Rehabilitation Hospital, which has convalescent rehabilitation wards [30] and employs a fall prevention system based on previous research [13, 31]. Fall prevention at this facility is implemented through a systematic process based on direct clinical assessment of patients’ actual mobility performance and observed behaviors. These assessments are conducted upon admission and are reevaluated as needed according to changes in functional status. Standardized mobility assessments [32, 33], together with multidisciplinary observation of patients’ activities of daily living (ADLs) on the ward, are used to determine the level of assistance required, appropriate mobility aids (e.g., cane or wheelchair), and permitted range of activity (e.g., whether the patient may ambulate independently within the room or ward, or requires supervision for toileting). Based on this structured assessment, color-coded wristbands are applied to indicate the prescribed activity level, and sensor-based monitoring is implemented when clinically indicated.

The study population comprised 824 consecutive patients with hip fractures admitted between January 1, 2015, and December 31, 2024. In accordance with the Declaration of Helsinki, the study was reported according to the STROBE guidelines. Owing to the study’s retrospective design, the requirement for informed consent was waived, and an opt-out method was applied.

### Data collection

Demographic and clinical data, including age, sex, and length of hospitalization, were retrieved from electronic medical records and hospital databases by the principal investigator. Fall events and fall-related fractures during hospitalization were identified through incident reports, which were completed by the medical staff who witnessed or found the event. A fall was defined as an event where a person unintentionally comes to rest on the ground, floor, or another lower level, in accordance with the World Health Organization criteria [34].

## Functional assessment

Motor and cognitive abilities were evaluated using the FIM [24]. The scale consists of 13 motor and five cognitive items scored from 1 (total dependence) to 7 (complete independence) [24, 35], with established reliability in hip fracture populations [36]. Assessments were routinely conducted at 2-week intervals by trained nursing staff. Linear interpolation was used to estimate values between assessments, following the same approach used in our previous work [29].

### Statistical analysis

Participants were categorized as fallers or non-fallers, and baseline characteristics were compared between the groups. Statistical comparisons were conducted using the unpaired t-test, Wilcoxon rank-sum test, or chi-squared test, as appropriate for the variable type and distribution.

Functional status was evaluated using FIM. FIM scores were assessed for each patient at admission, at 2-week intervals from the date of admission, and at discharge. Daily scores for the 18 FIM items (13 motor items and 5 cognitive items) were estimated for each patient using linear interpolation. After interpolation, daily motor scores and cognitive scores were assigned to each patient. The motor score was calculated as the mean of the 13 motor items, and the cognitive score as the mean of the five cognitive items.

First, to clarify the fall risk associated with each functional level, we calculated the fall rate by dividing the total number of falls by the aggregate person-days for each of the mean motor and mean cognitive FIM score categories (1–7 points). Second, to analyze the association between combinations of motor and cognition functional status and fall risk, we categorized the FIM motor and cognitive scores as follows: complete dependence (1–2 points), modified dependence (3–5 points), and independence (6–7 points), according to predefined criteria [37, 38]. This resulted in nine functional combinations (three motor by three cognitive categories). The primary outcome was the fall rate (per 1,000 person-days) for each combination, calculated by dividing the number of falls occurring within a specific combination by its corresponding aggregate person-days. The secondary outcomes for each combination included the total number of falls, their proportion relative to all falls, and the number of serious falls resulting in fractures. Observation periods (person-days) were accumulated for each functional combination across the entire hospitalization period. Because functional status changed over time, the data structure allowed individual participants to contribute person-days to multiple functional states. This analytical approach was used to examine the relationship between fall risk and the actual number of falls across functional states.

All data analyses were performed using STATA/BE 17 (StataCorp). P values < 0.05 were considered statistically significant.

## Results

Table 1 presents the characteristics of the 824 participants at admission and discharge. The mean age of the participants was 83.2 years (standard deviation, 7.2), with an age range of 65–101 years. Of these participants, 125 (15.2%) experienced at least one fall during hospitalization. Those who fell were older and had significantly lower FIM motor and cognitive scores at admission and discharge than those who did not. Throughout the hospitalization period, 182 falls were recorded over 56,476 person-days (average fall rate of 3.2/1,000 person-days). Among those who fell, 34 (27.2%) experienced multiple falls (two or more) during their hospital stay.

**Table 1:** Participants’ characteristics

	All (*n* = 824)	Non-faller (*n* = 699)	Faller (*n* = 125)	*P*-value
Sex, male/female, *n*	190/634	158/541	32/93	0.537
Age, years, mean (SD)	83.2 (7.2)	82.9 (7.3)	84.7 (6.7)	0.008
Length of hospital stay, days, mean (SD)	67.4 (23.0)	66.0 (23.3)	75.5 (19.6)	< 0.001
FIM at admission, median (IQR)				
Total score	70.0 (33.5)	70.0 (34.0)	63.0 (27.0)	0.002
Motor score	46.0 (24.0)	47.0 (24.0)	42.0 (18.0)	0.002
Cognitive score	24.0 (12.5)	25.0 (12.0)	22.0 (11.0)	0.005
FIM at discharge, median (IQR)				
Total score	103.0 (39.0)	104.0 (40.0)	94.0 (36.0)	< 0.001
Motor score	74.0 (27.0)	76.0 (27.0)	67.0 (24.0)	< 0.001
Cognitive score	28.0 (13.0)	28.0 (12.0)	25.0 (12.0)	< 0.001

*SD * standard deviation, *IQR * interquartile range, *FIM * Functional Independence Measure

**Fig. 1:**
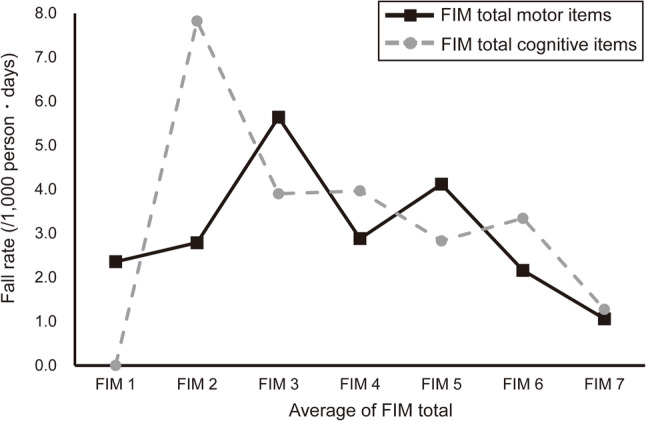
Falls rates by ability based on the motor and cognitive item scores on the FIM

Figure 1 illustrates the fall rates based on the mean FIM motor and cognitive items scores. The highest fall rate was 5.6/1,000 person-days for motor scores of 3 and 7.8/1,000 person-days for cognitive scores of 2. Both motor and cognitive scores showed a nonlinear, mountain-shaped pattern.

**Fig. 2:**
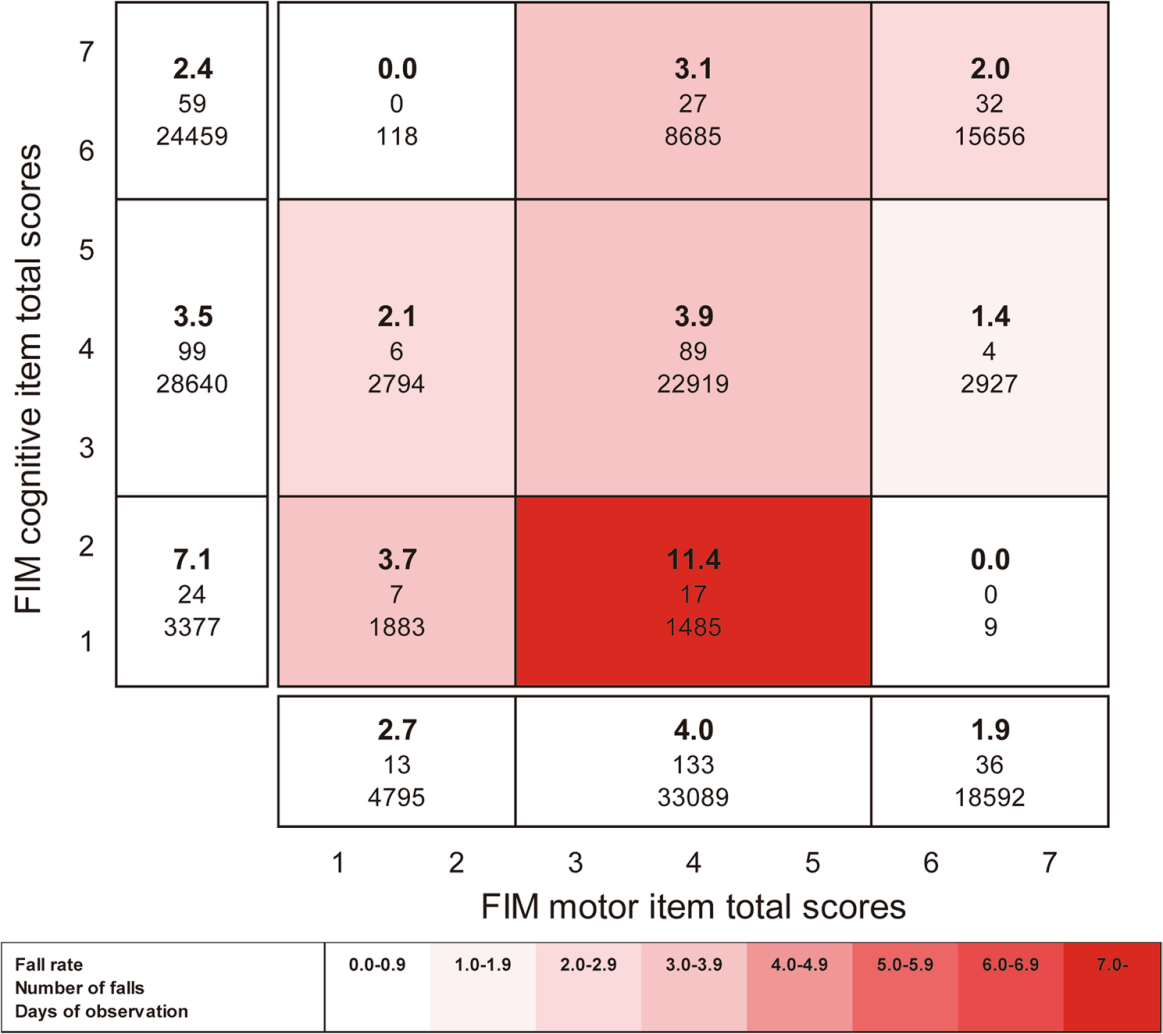
Falls rates according to motor and cognitive abilities. The risk of falls (fall rate) corresponding to various combinations of motor and cognitive abilities is shown. Colors indicate the degree of fall risk. The number of falls and observation days required to calculate the fall rate are also shown. FIM, Functional Independence Measure

Figure 2 shows the fall rate based on the combination of motor and cognitive functional states. The participants with modified dependence in the motor functional state and complete dependence in the cognitive functional state had the highest fall rates (11.4/1,000 person-days), followed by those with modified dependence in both motor and cognitive functional states (3.9/1,000 person-days) and those with complete dependence in motor and cognitive functional states (3.7/1,000 person-days). In contrast, the lowest fall rates were observed among those with independence in the motor functional state and modified dependence in the cognitive functional state (1.4/1,000 person-days), followed by those in both motor and cognitive independence states (2.0/1,000 person-days).

**Fig. 3:**
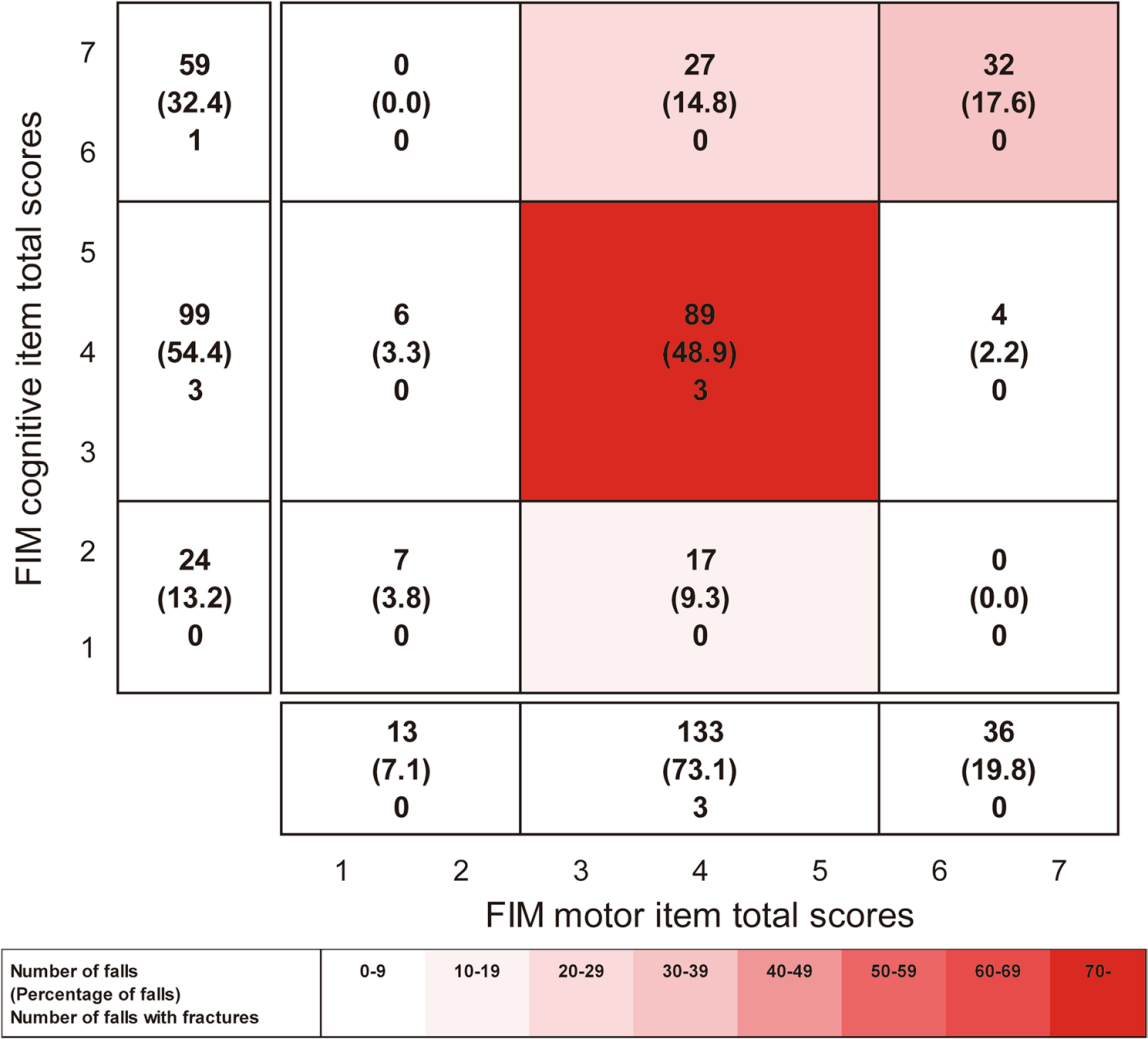
Number of falls according to motor and cognitive abilities. The number of observed falls is categorized according to different combinations of motor and cognitive abilities. Colors represent the number of falls. Additionally, it includes information on the percentage of falls relative to the total number of falls and the number of falls resulting in fractures. FIM, Functional Independence Measure

Figure 3 shows the number of falls, ratio of the number of falls to the total number of falls, and number of serious falls with fractures, based on the mean scores of the motor and cognitive FIM items. The highest number of falls, 89 falls (48.9%), occurred among those with modified dependence in motor and cognitive functions. Notably, despite having the highest fall rate, the combination of modified motor ability dependence and complete cognitive ability dependence accounted for only 17 falls (9.3% of total falls reported). In contrast, on excluding conditions in which no falls occurred, independence in both motor and cognitive states (the second lowest fall rate) represented 32 falls (17.6% of all reported falls), i.e., approximately 1.9 times the number of falls observed in the highest fall-rate state.

## Discussion

In this study, we determined the relationship between fall frequency during hospitalization and fall risk based on motor and cognitive functional states in patients with hip fractures. Notably, even within this population that is widely recognized as being at high risk for falls, we were able to clearly identify distinct conditions of extremely high risk and relatively low risk based on changes in functional status. Specifically, excluding functional states in which no falls occurred, the fall rate differed by as much as eight-fold between the highest-risk state (modified dependence in motor ability and complete dependence in cognitive ability) and the lowest-risk state (independence in motor ability and modified dependence in cognitive ability). Another important finding was that the state associated with the highest fall risk accounted for only 9.3% of all falls. This finding supports our hypothesis that the state with the highest fall risk does not necessarily correspond to the functional state associated with the highest number of falls.

In previous studies, 12–39% of the patients with fall-related injuries admitted to rehabilitation hospitals experienced a fall during hospitalization [3–5]. In our study, 15.2% of the patients experienced a fall during hospitalization, consistent with the results reported in previous studies [3, 4]. Traditionally, fall prevention programs have focused on identifying patients at high risk and directing preventive resources toward these individuals [9–11, 39, 40]. However, our findings suggest that this strategy alone may be insufficient to reduce the overall burden of falls. In this study, the fall risk was analyzed using FIM scores and their temporal changes during hospitalization. Motor function demonstrated a nonlinear, “inverted U-shaped” association with fall risk, with the highest risk occurring at moderate levels of function. Similar findings have been reported for both geriatric ward settings [41] and patients with stroke [29]. This pattern is clinically reasonable: functional states with severe impairment often preclude activities that lead to falls, whereas states of high functional independence allow for safer movement. Cognitive function also demonstrated an inverse U-shaped relationship with the fall risk. The highest fall risk was observed during the state of severe cognitive dependence combined with moderate motor dependence, suggesting that cognitive impairment alone does not necessarily increase fall risk unless accompanied by some mobility. Importantly, when motor and cognitive functional states were combined, the state of modified motor dependence consistently appeared in the top two highest-risk groups, highlighting motor function—not cognitive function—as the primary determinant of fall risk in patients with hip fractures. Previous qualitative research supports this, indicating that patients often challenge their abilities because of motivation, confidence, or overestimation of capability, even when cognitive function is largely intact [42].

The overall average fall rate during hospitalization in this study population was 3.2 falls per 1,000 patient days. However, when analyzed by functional status and on excluding states where no falls were observed, the fall rate showed a clear gradient ranging from 1.4 to 11.4 falls per 1,000 patient days. The lowest fall rate (1.4 falls per 1,000 patient days) was comparable to that reported for community-dwelling older individuals [43, 44], whereas the highest-risk state reached 11.4 falls per 1,000 patient days, showing a marked difference. In other words, even within the patient population with hip fractures, generally considered to have a high fall risk, the fall risk markedly differed according to functional states that changes during hospitalization. These findings suggest that using FIM categorization based on relatively simple measures of motor and cognitive function can identify significant differences in fall risk.

In contrast, the states with the highest fall risk did not necessarily correspond to those with the highest number of falls. A substantial number of falls were observed even among those with relatively independent functional states when the fall risk was low. These discrepancies arise because fall frequency is influenced not only by the level of fall risk but also by the amount of time patients spend in each functional state [29, 45, 46]. Consequently, a prolonged stay in a specific functional state increases the cumulative opportunity for a fall to occur. This implies that even states with a seemingly low fall risk (comparable to that of community-dwelling older adults [43, 44]) must be targeted for intervention to effectively reduce the hospital’s overall fall volume. Herein lies the structural dilemma: whether to concentrate limited resources on “high-risk individuals” or to distribute them across “high-volume groups,” where individual risk is relatively lower but the total number of observed falls is higher.

In a previous study of patients with stroke that investigated fall risk and the number of falls based on the same combination of FIM motor and cognitive categories used in the present study [29], the highest fall risk was found in the functional state of modified motor dependence combined with complete cognitive dependence. Interestingly, despite the different disease, the same category showed the highest fall risk. Furthermore, surprisingly, the fall rate associated with this functional state was 10.8 falls per 1,000 person-days, which was comparable to the highest fall rate observed in the present hip fracture group (11.4 falls per 1,000 person-days). These findings suggest that functional status alone may allow some estimation of the level of fall risk, regardless of diseases.

The clinical implications of this study are twofold. First, we identified a clear gradient in fall risk associated with dynamic changes in functional status during hospitalization, even in a group considered uniformly high-risk, such as patients with hip fractures. By combining motor and cognitive FIM scores, we confirmed that fall risk fluctuates approximately eight-fold (1.4–11.4 falls per patient day) across different functional states. In particular, the functional state characterized by moderate motor dependence and complete cognitive dependence exhibited the highest risk. For patients at this specific phase, intensive and immediate interventions—such as enhanced staff supervision and monitoring via bed sensors—are expected to contribute to fall prevention. Second, this study highlighted a structural dilemma in fall prevention strategies: merely identifying “high-risk states” is insufficient. Our real-world clinical data demonstrated that while falls in the highest-risk state accounted for only 9.3% of all events, a substantial number of falls occurred even in states considered relatively low risk. Community-based prospective studies have reported that even older adults classified as having “no fall risk” experience falls and fall-related injuries [47]. Taken together, these findings suggest that fall events are not limited to the highest-risk states. Therefore, effective fall management requires a dual strategy that addresses both the quality and quantity of risk: implementing immediate preventive measures for high-risk states, while simultaneously employing comprehensive bottom-up approaches—such as environmental modifications and staff and patient education—to counteract the cumulative risk associated with prolonged observation periods in lower-risk states.

This study has some limitations. First, its retrospective, single-center design limits the generalizability of the findings to other institutions with different fall prevention strategies. Furthermore, falls occurred under existing hospital-based fall prevention measures, and this may have reduced the overall incidence of falls and potentially attenuated differences among functional states. Second, fall events were identified through incident reports. While widely used, this method can lead to underreporting or misclassification, which may affect the accuracy of the calculated fall rates [48, 49]. Finally, while the FIM-based categories identified risk states, incorporating additional risk factors such as age, severity of motor impairment, and medication use may enable more detailed stratification. Future research should investigate how these variables interact with functional states to further refine risk prediction. Nevertheless, this study suggests that even without these additional factors, FIM-based categories alone may serve as a practical tool for identifying fall risk during dynamic functional changes.

## Conclusions

This study demonstrated that fall risk fluctuates dynamically in patients with hip fractures according to changes in their functional status during hospitalization. In particular, the fall risk peaked during the functional states characterized by a combination of modified motor dependence and complete cognitive dependence, with a fall rate approximately eight times higher than that of the lowest-risk state. Conversely, the total number of falls was higher in states considered relatively low risk than in the highest-risk state. This finding highlights a structural dilemma: the extended observation periods in specific functional states increases cumulative opportunities for falls. Therefore, effective fall management requires a dual approach: intensive immediate interventions to reduce risk during high-risk stages and comprehensive prevention strategies (such as patient education and environmental modifications) to reduce total falls throughout the entire hospitalization period. Such strategies are essential to address both the intensity of individual risks and total volume of falls.

## Data Availability

The datasets analyzed during the current study are available from the corresponding author upon reasonable request.

## Abbreviations

ADL: Activities of daily living
FIM: Functional independence measure
